# Poster Session I - A38 MEGACYSTIS-MICROCOLON-INTESTINAL HYPOPERISTALSIS SYNDROME (MMIHS): A RARE CASE WITH FULL CLINICAL EXPRESSION

**DOI:** 10.1093/jcag/gwaf042.038

**Published:** 2026-02-13

**Authors:** S Alobaidan, M Sherlock, E Ratcliffe, M Livingston, A Mukerji, N Lepore

**Affiliations:** Department of Pediatric Gastroenterology & Nutrition, McMaster University., Hamilton, ON, Canada; Department of Pediatric Gastroenterology & Nutrition, McMaster University., Hamilton, ON, Canada; Department of Pediatric Gastroenterology & Nutrition, McMaster University., Hamilton, ON, Canada; Department of Pediatric Surgery, McMaster University, Hamilton, ON, Canada; Neonatal Intensive Care Unit, McMaster University, Hamilton, ON, Canada; Department of Pediatric Gastroenterology & Nutrition, McMaster University., Hamilton, ON, Canada

## Abstract

**Background:**

Megacystis Microcolon Intestinal Hypoperistalsis Syndrome (MMIHS) is a rare congenital disorder characterized by a triad of dilated urinary bladder (megacystis), microcolon, and intestinal hypoperistalsis due to myopathic dysfunction of the bowel and bladder. Mutations in genes such as *ACTG2* and *MYH11* have been implicated. Although the condition was historically fatal, advances in intestinal rehabilitation and transplantation have improved survival and outcomes

**Aims:**

Presentation of a rare case of MYH11-related syndrome with full clinical features, and to expound its potential clinical implications, diagnostic challenges, and implications of multidisciplinary care

**Methods:**

Case report and literature review

**Results:**

An eight-month-old girl was born at 37 weeks’ gestation to consanguineous parents. Family history revealed similar findings in two pregnancies of a maternal aunt and one prior pregnancy of the mother, all undiagnosed. Antenatal ultrasound at 21 weeks was suggestive of duodenal atresia, renal pelvic dilation, and megacystis with polyhydramnios. Amniocentesis and whole-exome sequencing confirmed a homozygous pathogenic MYH11 variant (c.3424C>T; p.R1142*), diagnostic of autosomal recessive MMIHS.

Baby was born with APGAR scores (3-6-7) and was intubated immediately. Urinary catheter was inserted at 6 hours of age due to anuria. Imaging confirmed duodenal stenosis, absent peristalsis, microcolon and severe bilateral hydronephrosis. The patient underwent multiple surgical procedures. Initial exploration at six days of age revealed multiple intestinal stenoses and malrotation, and an end jejunostomy and mucous fistula were created. Due to persistent functional obstruction further exploration revealed the bowel to have multiple stenotic, but patent segments. There was no peristalsis. Her colon contained thick mucous plugs. A PEG tube was placed.

Her urological course was marked by grade IV vesicoureteral reflux and recurrent urinary tract infections despite prophylactic antibiotics, requiring a vesicostomy at 6 months of age to relieve bladder distension and to facilitate drainage. She currently has CKD stage 2, Cystatin C (GFR 70 ml/min/1.73 m^2^). She remains dependent on total parenteral nutrition, with weight tracking at the 3rd centile and stable liver function

**Conclusions:**

This case highlights the complexity of MMIHS and the importance of early, ideally prenatal, recognition. Despite advances in intestinal rehabilitation and transplantation improving survival, optimal outcomes still require intensive multidisciplinary care and individualized planning

**Funding Agencies:**

None

